# The Impact of NAD Bioavailability on DNA Double-Strand Break Repair Capacity in Human Dermal Fibroblasts after Ionizing Radiation

**DOI:** 10.3390/cells12111518

**Published:** 2023-05-31

**Authors:** Maria Svetlova, Ljudmila Solovjeva, Andrey Kropotov, Andrey Nikiforov

**Affiliations:** Institute of Cytology, Russian Academy of Sciences, St. Petersburg 194064, Russia; mila.solovjeva@gmail.com (L.S.); a.kropotov@gmail.com (A.K.)

**Keywords:** DNA double-strand break repair, ionizing radiation, nicotinamide adenine dinucleotide, human dermal fibroblasts

## Abstract

Nicotinamide adenine dinucleotide (NAD) serves as a substrate for protein deacetylases sirtuins and poly(ADP-ribose) polymerases, which are involved in the regulation of DNA double-strand break (DSB) repair molecular machinery by various mechanisms. However, the impact of NAD bioavailability on DSB repair remains poorly characterized. Herein, using immunocytochemical analysis of γH2AX, a marker for DSB, we investigated the effect of the pharmacological modulation of NAD levels on DSB repair capacity in human dermal fibroblasts exposed to moderate doses of ionizing radiation (IR). We demonstrated that NAD boosting with nicotinamide riboside did not affect the efficiency of DSB elimination after the exposure of cells to IR at 1 Gy. Moreover, even after irradiation at 5 Gy, we did not observe any decrease in intracellular NAD content. We also showed that, when the NAD pool was almost completely depleted by inhibition of its biosynthesis from nicotinamide, cells were still able to eliminate IR-induced DSB, though the activation of ATM kinase, its colocalization with γH2AX and DSB repair capacity were reduced in comparison to cells with normal NAD levels. Our results suggest that NAD-dependent processes, such as protein deacetylation and ADP-ribosylation, are important but not indispensable for DSB repair induced by moderate doses of IR.

## 1. Introduction

Among various DNA lesions, double-strand breaks (DSB) are the most dangerous for the cell because they can lead to chromosome rearrangements, oncogenic transformation or cell death if not repaired [1]. DSBs are repaired by two major pathways: homologous recombination (HR) and non-homologous end joining (NHEJ) [2,3]. The key steps of HR include the generation of 3’ single strands by resection of broken DNA ends with nucleases and RAD51-dependent invasion of 3’ single-stranded DNA into a homologous duplex that is available only during S and G2 phases of the cell cycle [2]. In mammalian cells, NHEJ is the major pathway of DSB repair, acting by direct sealing of DSB ends in all phases of the cell cycle. NHEJ is mediated by DSB sensing heterodimer Ku70/Ku80, which recruits and activates the catalytic subunit of DNA-dependent protein kinase (DNA-PKcs) at DSB sites followed by ligation using DNA ligase IV/XRCC4/XLF complex [3]. The central player of DNA damage response to DSB is ataxia-telangiectasia mutated kinase (ATM). ATM is rapidly recruited to the site of DSB through interaction with DSB recognition complex MRE11/RAD50/NBS1 (MRN), which results in ATM activation by autophosphorylation at Ser1981 [4,5]. Activated ATM phosphorylates multiple downstream targets promoting DNA repair and activating cell-cycle arrest [6]. One of the earliest events of cellular response to DSB is ATM-dependent phosphorylation of histone H2AX at serine 139 (referred to as γH2AX) at the sites of DNA damage [7,8]. In addition to ATM, two other members of the phosphatidylinositol 3-kinase-related kinase family regulating DNA damage response in mammalian cells, DNA-PK and ATR (ATM- and Rad3-related) have been shown to phosphorylate H2AX at the sites of DSB [9,10,11]. γH2AX spreads across large chromatin domains surrounding DSB (up to 2 Mb) [12,13] and plays an important role in the regulation of HR and NHEJ in mammalian cells [14]. These DSB-induced γH2AX domains are visualized as foci by immunofluorescence microscopy. It has been demonstrated that the number of γH2AX foci directly correlates to the number of DSB [13,15,16]. Therefore, the quantification of γH2AX foci is widely used to monitor DSB formation and repair [17].

Nicotinamide adenine dinucleotide (NAD) is an essential coenzyme that mediates redox reactions in central metabolic pathways. NAD also serves as a substrate for several families of regulatory proteins, such as class III protein deacetylases (sirtuins, SIRTs), mono-ADP-ribosyltransferases, and poly(ADP-ribose) polymerases (PARPs), that control vital cell processes including DNA damage response and DNA repair [18,19,20]. There is increasing evidence that NAD-dependent protein ADP-ribosylation and deacetylation are involved in the promotion of DSB repair through various mechanisms [21,22].

It has recently been demonstrated that PARP1, the best-characterized member of the PARP family, serves as a sensor of DSB along with Ku70/Ku80 and MRN complexes [23]. PARP1 is rapidly recruited to DSB sites and activated by binding to free DNA ends. Upon activation, using NAD as a substrate, PARP1 attaches polymers of ADP-ribose (PAR) to itself or a variety of histone and non-histone target proteins. This leads to local chromatin relaxation and recruitment of chromatin remodeling factors and DSB repair proteins involved in HR and NHEJ (reviewed in [24]). For instance, it has been shown that PARP1 interacts with the MRN complex and stimulates its recruitment to the sites of DSB [25]; ATM kinase interacts with PAR [26], and this interaction stimulates its activity [27,28]; BRCA1, which is involved in DSB end resection and RAD51 loading onto DNA, is targeted to DSB by binding of BRCT motifs of BARD1, a heterodimeric partner of BRCA1, to PAR [29]; PARP1 can form a complex with Ku70/Ku80 [30] and DNA-PKcs [31], whereas poly-ADP-ribosylation of DNA-PKcs stimulates its kinase activity [32]. Moreover, it has been shown that PARP1 recruitment protects DSB-free ends from nucleases, thereby negatively regulating DNA end resection and directing repair to the NHEJ pathway [33]. NHEJ can also be promoted by PARP1-dependent recruitment of chromatin structure modulator CHD2, which then facilitates the assembly of NHEJ repair complexes at DNA breaks [34]. Another member of the PARP family, mono ADP-ribosyltransferase PARP3, has been demonstrated to accelerate the NHEJ pathway of DSB repair by facilitating the retention of XRCC4/LIG4 complex at DNA damage sites [35]. PARP3 also ADP-ribosylates the Ku70/Ku80 complex and facilitates its recruitment to DSB. Furthermore, PARP3, together with Ku70/Ku80 complex, limits DSB end resection, thus defining the choice between NHEJ and HR pathways [36].

NAD-dependent protein deacetylases sirtuins have also been shown to play an important role in DSB repair. SIRT1, SIRT6, and SIRT7, members of the sirtuin family that are mainly localized in the nucleus, deacetylate a number of proteins involved in HR and NHEJ repair leading to their activation and/or recruitment to the sites of DNA damage (reviewed in [21]). SIRT1 and SIRT6 are rapidly recruited to DSB [37,38], and their depletion impairs early events of DNA damage response, such as ATM-dependent phosphorylation of H2AX [37,39]. SIRT1 recruitment to DSB is dependent on ATM. On the other hand, SIRT1 stimulates autophosphorylation and activation of ATM, thereby stabilizing ATM at DSB sites, demonstrating a synergistic relationship between these two DNA repair proteins [38]. SIRT1 maintains NBS1 in a hypoacetylated state facilitating its phosphorylation by ATM in response to IR [40] and also can promote DSB repair by deacetylation of Ku70 [41], DNA helicase WRN [42], and transcriptional corepressor KAP1 [43]. SIRT6, having deacetylase and mono-ADP-ribosyltransferase activity, promotes both the HR and the NHEJ pathways of DSB repair [37,44,45]. SIRT6 primarily functions as a DSB sensor. It binds directly to the DSB and initiates DNA damage response [45]. SIRT6 has been shown to mono-ADP-ribosylate PARP1, thereby stimulating its activity and enhancing DSB repair [44]. SIRT6 also interacts with and stabilizes DNA-PK at DSB sites [46] and recruits chromatin remodeler SNF2H, increasing the accessibility of DNA repair factors to damaged DNA [37]. In contrast to SIRT1 and SIRT6, SIRT7 is recruited to the sites of DSB with slow kinetics in a PARP1-dependent manner [47]. SIRT7-dependent deacetylation of H3K18ac has been shown to be important for NHEJ promotion [48]. SIRT7 also deacetylates ATM kinase at K3016, and this step is a prerequisite for ATM dephosphorylation and deactivation at the late stage of DSB repair [49].

Protein deacetylation and ADP-ribosylation, implemented by sirtuins and PARPs in response to DSB induction, are supposed to be controlled by intracellular NAD levels since these reactions are accompanied by the cleavage of NAD to nicotinamide (Nam) and ADP-ribose. However, the impact of NAD bioavailability on DSB repair remains poorly characterized.

In this study, using γH2AX foci quantification as an indirect detection of DSB, we have examined how pharmacological modulation of NAD levels affects the efficiency of DSB repair in human dermal fibroblasts (HDF) after exposure to ionizing radiation (IR). We have demonstrated that stimulation of NAD biosynthesis by nicotinamide riboside (NR) significantly increases the level of intracellular NAD, but this does not influence the DSB repair capacity in HDF after exposure to moderate doses of IR. Moreover, we have not observed any depletion of the NAD pool during the DNA damage response induced by IR at a dose of 1 or 5 Gy. We also have shown that critical depletion of the NAD in HDF by inhibition of NAD biosynthesis from Nam impairs the IR-induced activation of ATM kinase and its colocalization with γH2AX and decreases DSB repair capacity.

## 2. Materials and Methods

### 2.1. Materials

Unless otherwise specified, all chemicals and reagents were of analytical grade and were purchased from Sigma-Aldrich (St. Louis, MO, USA) and Amresco (Solon, OH, USA). NR was a kind gift from Prof. Marie Migaud (Mitchell Cancer Institute, University of South Alabama, Mobile, AL, USA). Cell culture reagents and lab plasticware were from Gibco (Waltham, MA, USA), Greiner Bio-One (Monroe, NC, USA), and Orange Scientific (Braine-l’Alleud, Belgium). The ultrapure water was obtained from a Milli-Q Synthesis purification system (Millipore, Burlington, MA, USA). The following antibodies were used: rabbit anti-γH2AX (phospho S139) (abcam, Cambridge, UK, ab81299), mouse monoclonal anti-Ki-67 (Thermo Fisher Sci., Waltham, MA, USA, MA1-2020), mouse monoclonal anti-phospho-ATM (S1981) (abcam, Cambridge, UK, ab36810), secondary antibodies: Alexa Fluor 488-conjugated goat anti-rabbit IgG, Alexa Fluor 568-conjugated goat anti-mouse IgG, Alexa Fluor 568-conjugated goat anti-rabbit IgG, and Alexa Fluor 488-conjugated goat anti-mouse IgG (Molecular Probes, Eugene, OR, USA, A11008, A11004, A11011, A11001). Click-iT Plus EdU imaging kit was obtained from Invitrogen (Waltham, MA, USA, C10086).

### 2.2. Cell Culture and Ionizing Radiation Treatment of Cells

Human dermal fibroblasts (HDF) (purchased from Pokrovsky Stem Cell Bank, St. Petersburg, Russia) were cultured in Minimum Essential Medium (MEM) supplemented with 10% fetal bovine serum (FBS), 100 U/mL penicillin, 100 μg/mL streptomycin, and 2 mM glutamine. The cells were cultured at 37 °C in a humidified atmosphere of 5% CO_2_. NR (150 µM) or FK866 (2 µM) were added to the culture medium as indicated. Cells were exposed to ionizing radiation (IR) at 1 or 5 Gy using X-ray irradiator RAP-150/300-14 (Promrentgen, Moscow, Russia) with a Cu/Al filter.

### 2.3. Flow Cytometry

In total, 2.5 × 10^5^ HDF cells were plated in 6-well plates. After 1, 2, 3, 4, and 7 days, cells were trypsinized and stained with 50  μg/mL propidium iodide for the estimation of the number of dead and viable cells. Flow cytometry was performed using a CytoFLEX instrument (Beckman Coulter Inc., Brea, CA, USA). Analysis was carried out using CytExpert 2.0 Software (Beckman Coulter Inc., Brea, CA, USA).

### 2.4. NAD Quantification

Intracellular NAD content was determined by a colorimetric enzymatic assay using a NAD/NADH Quantification Kit (Sigma-Aldrich, St. Louis, MO, USA, Cat. No. MAK037). A total of 2.5–3.0 × 10^5^ cells/well were seeded in 12-well plates. The next day, the cells were lysed directly in wells with 200 μL extraction buffer provided in NAD/NADH Kit, and further procedures were performed according to the manufacturer’s protocol. NAD concentration was obtained by normalizing the measured NAD content to the protein amount in the sample and was expressed as pmol/μg protein. Protein concentration was determined using Pierce ^TM^ BCA Protein Assay Kit (Thermo Scientific, Rockford, IL, USA).

### 2.5. MTT-Assay

Cell metabolic activity was determined using a 3-(4,5-dimethylthiazol-2-yl)-2,5-diphenyltetrazolium bromide (MTT) assay kit (Rosmedbio, St Petersburg, Russia). The cells were seeded in 24-well plates (0.3 × 10^5^ cells/well) and incubated for 1–7 days in a CO_2_ incubator in a humidified atmosphere with 5% CO_2_ at 37 °C. An MTT solution in PBS was added to the growth medium with a final concentration of 0.5 mg/mL, and cells were incubated for 3 h in a CO_2_ incubator. After that, the growth medium was discarded, and 1 mL DMSO was added to each well. The plate was incubated with shaking at 42 °C for 30 min until the complete solubilization of purple formazan crystals. Spectrophotometric absorbance of samples was measured at 570 nm wavelength using Multiskan ^TM^ FC Microplate Photometer (Thermo Fisher Scientific, Waltham, MA, USA).

### 2.6. Immunostaining of γH2AX and Ki-67 Combined with the Detection of S-Phases after EdU Incorporation

Cells were grown on 18 × 18 mm glass coverslips placed in Petri dishes. After IR, the cells were incubated for 1–24 h in a growth medium. In addition, 5-ethynyl-2′-deoxyuridine (EdU) (10 μM) was added 20 min before fixation for labeling of S-phase cells. The cells were fixed with 4% formaldehyde at +4 °C, rinsed with PBS, permeabilized with 0.5% Triton X-100 in PBS for 15 min with shaking, rinsed with 3% BSA in PBS, and then, copper-catalyzed click reaction of EdU with Alexa Fluor 647 picolyl azide was performed in accordance with the Click-iT Plus EdU imaging kit manufacturer’s recommendations. After blocking in 1% Blocking Reagent (Roche, Mannheim, Germany) in PBS with 0.02% Tween 20 for 30 min at 37 °C, immunofluorescence staining for detection of γH2AX, the marker of DSB repair, and Ki-67, the marker of cell proliferation [50,51], was performed. The dilution of antibodies was carried out in 0.5% Blocking Reagent in PBS with the addition of 0.02% Tween 20. All incubations with antibodies were performed at 37 °C. Between subsequent incubations, slides were washed with shaking for 30 min in PBS supplemented with 0.1% Tween 20. Cells were incubated for 1 h with the following primary antibodies: rabbit anti-γH2AX (1:100) combined with monoclonal mouse anti-Ki-67 (1:50), and 40 min with secondary antibodies: Alexa Fluor 488-conjugated goat anti-rabbit IgG and Alexa Fluor 568-conjugated goat anti-mouse IgG (1:400). DNA was counterstained for 10 min at room temperature with 0.5 μg/mL 4′,6-diamidino-2-phenylindole (DAPI) (Sigma-Aldrich, St. Louis, USA) in PBS, and samples were mounted in a Citifluor antifade solution AF1 (Science Services, Munich, Germany).

### 2.7. Immunostaining of γH2AX and Phospho-ATM Combined with the Detection of S-Phases after EdU Incorporation

For γH2AX/phospho-ATM (S1981) (pATM) double immunostaining, Ki-67 marker was not used. Irradiated cells were incubated with EdU (10 µM) 20 min before fixation. Cell fixation, permeabilization, washing, blocking, detection of EdU with Alexa Fluor 647 picolyl azide, and other protocol details were the same as in Section 2.6. After EdU detection, cells were stained with primary rabbit anti-γH2AX antibody (1:100) and mouse monoclonal anti-phospho-ATM (S1981) antibody (1:100) and 40 min with secondary antibodies: Alexa Fluor 488-conjugated goat anti-mouse IgG and Alexa Fluor 568-conjugated goat anti-rabbit IgG (1:400). DNA was counterstained with DAPI in PBS, and samples were mounted in the Citifluor antifade solution.

### 2.8. Confocal Microscopy and Image Acquisition

Images were acquired using a confocal Leica TCS SP5 microscope (Leica Microsystems, Wetzlar, Germany) equipped with HCX PL APO 40×/1.25 oil immersion objective, 488 nm argon, 543 nm HeNe, 633 nm HeNe, and 405 nm diode lasers. Leica LAS X 3.0.0.15697 software (Leica Microsystems, Wetzlar, Germany). Leica Microsystems, Wetzlar, Germany) was used for image analysis. Confocal sections were collected with the step size 0.71 μm, and maximum intensity projections of Z-stacks were obtained. In a single confocal section, voxel size was 189.6 nm × 189.6 nm × 713.3 nm (zoom 2, used for counting γH2AX and pATM foci) and 42.1 μm × 42.1 μm × 713.3 nm (zoom 9, used for colocalization analysis of γH2AX/pATM foci). Image size was 1024 × 1024 pixels.

### 2.9. Quantification of γH2AX and pATM Foci

Quantification of γH2AX foci after IR was performed only in G0 and G1 cells in images of HDF cells after Ki-67 immunostaining and EdU detection. Ki-67 labels cells in G1, S, G2, and M phases, and only resting (G0) cells are Ki-67 negative. Ki-67 positive EdU-incorporated S-phase cells were excluded from the analysis. G2 cells were excluded due to the increased size of the nucleus and the specific pattern of Ki-67 nuclear distribution (1 or 2 large and bright nucleoli). For γH2AX/pATM double immunostaining, EdU-incorporated S-phase cells were excluded from analysis, and G2 cells were excluded due to the increased size of the nucleus. For quantification of γH2AX and pATM foci number and the total projected area of foci per nucleus (referred to as “the total area of foci per nucleus” in the text), the IPLab v3.65 program (Scanalytics, Inc., Vernon, WI, USA) was used. Green channel images were converted to a grey scale, and the “Segmentation function” was applied to discriminate the foci from the background noise. The same thresholds of segmentation were applied in each series of experiments. The total area of foci per nucleus was measured in pixels.

### 2.10. Colocalization Analysis of γH2AX and pATM Foci

The images of central confocal sections of the nuclei captured at zoom 9 were taken for colocalization analysis of γH2AX and pATM foci. The analysis of colocalization was performed using the ImageJ 1.43 program (NIH, Bethesda, MD, USA). Image segmentation based on the “Difference of Gaussians” approach was performed using the GDSC Image J plugin in green (pATM) and red (γH2AX) channels separately for the elimination of background noise. Colocalization of segmented images of γH2AX and pATM foci was quantified by Pearson’s correlation using the “Manders’ coefficients” plugin as described in [52]. Pearson’s correlation coefficient (PCC) is a commonly used parameter for measuring the extent of foci overlap in image pairs. PCC is not sensitive to differences in pixel intensities in two different color channels and can vary in the range from −1 to +1. The more PCC, the higher extent of foci overlap (colocalization) [53]. PCC was obtained for 25 cells in each series of experiments.

### 2.11. Statistical Analysis

Statistical analysis was performed using SigmaPlot 12.5 (Systat Software Inc., San Jose, CA, USA). Differences between groups were analyzed using one- (or two-)way ANOVA with Tukey post hoc test. *p*-values < 0.05 were considered to be significant.

## 3. Results

### 3.1. Modulation of NAD Biosynthesis in Human Dermal Fibroblasts

**Figure 1:**
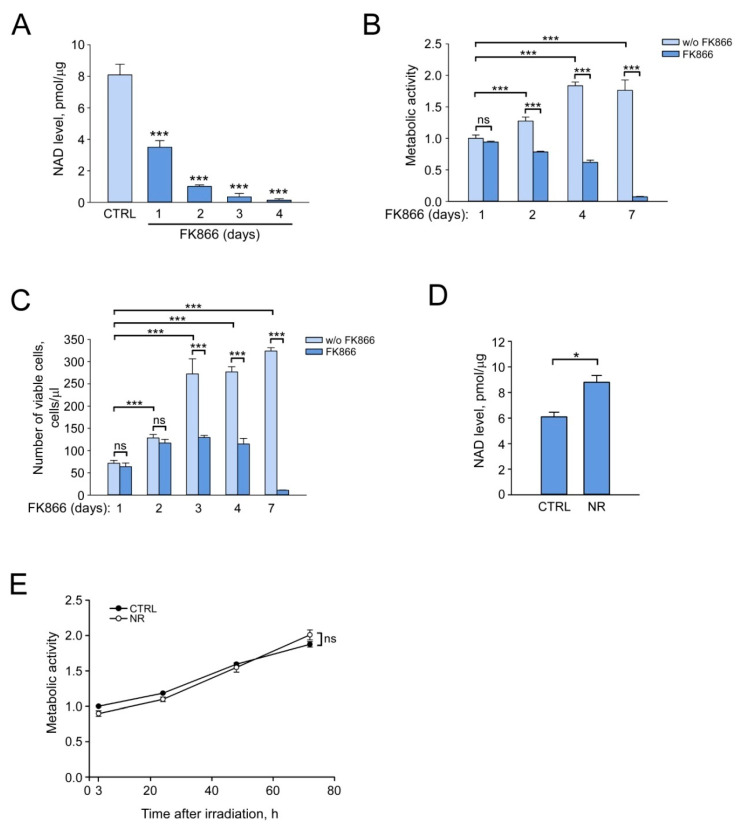
Pharmacological suppression and stimulation of NAD biosynthesis in human dermal fibroblasts. Human dermal fibroblasts (HDF) were cultured in Minimum Essential Medium (MEM) containing Nam, supplemented with 10% fetal bovine serum. (**A**–**C**) To inhibit NAD synthesis from Nam, cells were treated with FK866 for the indicated time periods. (**D**,**E**) To stimulate NAD synthesis, cells were treated with nicotinamide riboside (NR) for 24 h. (**A**,**D**) Intracellular NAD level measured by enzymatic colorimetric assay is expressed in picomoles per microgram of total protein in cell extracts. (**B**,**E**) Relative metabolic activity of cells was assessed using the MTT (3-(4,5-dimethylthiazol-2-yl)-2,5-diphenyltetrazolium bromide) assay. Metabolic activity of untreated cells (control) was taken as 1. (**C**) Cell proliferation was estimated by flow cytometry. Data are presented as mean ± standard deviation (SD) (*n* = 3). Statistical analysis of differences between the groups was carried out by one-way ANOVA with post hoc comparisons using Tukey test. *** indicates statistically significant difference at *p* < 0.001, * indicates statistically significant difference at *p* < 0.05, and ns indicates that statistical difference is not significant. CTRL marks untreated control cells.

To assess how changes in intracellular NAD levels can affect the efficiency of DNA DSB repair, we optimized the conditions for stimulation and suppression of NAD synthesis in human dermal fibroblasts (HDF). Cells cultured in the standard medium can synthesize NAD from the pyridine base Nam, a form of vitamin B3, via the salvage pathway. Nam is converted by the Nam phosphoribosyltransferase (NAMPT) to the Nam mononucleotide (NMN), which, in turn, is adenylated by NMN adenylyltransferases (NMNAT) to form NAD (Figure S1) [20,54]. For the suppression of NAD synthesis from Nam, we used FK866, a highly specific noncompetitive inhibitor of NAMPT [55]. The cells were cultured in the growth medium supplemented with 10% FBS in the presence of FK866 at a concentration of 2 µM during various periods of time. We also assessed cell viability by their metabolic activity, which we estimated by MTT assay, and cell proliferation, which was assessed by flow cytometry as described in Materials and Methods. One day after the addition of FK866 to HDF cells, the NAD level was about 45% of that evaluated in control cells (Figure 1A). At the same time, both metabolic activity (Figure 1B) and cell proliferation (Figure 1C) remained at the control level. During subsequent incubation with FK866, the concentration of intracellular NAD continued to fall, which was accompanied by suppression of both metabolic activity and cell proliferation (Figure 1A–C). After 4 days of incubation with FK866, the NAD level was nearly undetectable (Figure 1A), while the cells were still viable (Figure 1B,C). Seven days of treatment with NAMPT inhibitor resulted in cell death (Figure 1B,C).

It is important to note, that, besides Nam, another potential NAD precursor, tryptophan (at a concentration of 80 μM), is present in the growth medium. NAD can be synthesized from this precursor via the de novo pathway [20,54] (Figure S1). Our data indicate that the de novo NAD synthesis in HDF is inactive or insufficiently active to maintain the physiological level of NAD when the synthesis of this dinucleotide from Nam is inhibited.

Furthermore, in order to increase the concentration of intracellular NAD, we used NR, the nucleoside form of vitamin B3. NR stimulates an alternative NAD biosynthesis pathway via its phosphorylation by NR kinases (NRK) to yield NMN [56] (Figure S1), and is currently one of the most widely used NAD boosting agents in various cellular and animal experimental models [57,58]. HDF were cultured in the presence of NR (at a concentration of 150 µM) for 24 h, then the concentration of NAD in the cell extract was determined. Stimulation of NAD synthesis by NR resulted in a significant increase (more than 40%) in NAD level compared to control cells (Figure 1D). At the same time, the presence of NR in the growth medium had no effect on the metabolic activity of irradiated cells (Figure 1E).

### 3.2. Stimulation of NAD Biosynthesis Does Not Affect γH2AX Foci Formation and Elimination in IR-Exposed HDF

**Figure 2:**
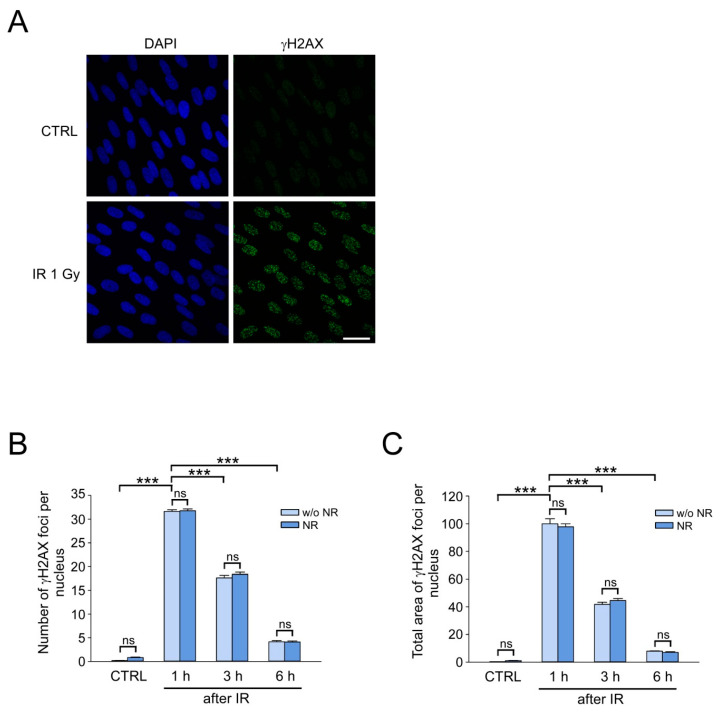
The effect of the stimulation of NAD biosynthesis on γH2AX foci formation and elimination in IR-exposed HDF. HDF were treated with ionizing radiation (IR) at a dose of 1 Gy. Cells were fixed at different time points after IR (as indicated) and stained for γH2AX. (**A**) γH2AX foci 1 h after IR visualized by immunofluorescence (green). Cell nuclei were counterstained with DAPI (blue). Scale bar, 30 µm. (**B**,**C**) Cells were pretreated with NR for 24 h before IR. NR was also present in the culture medium after IR. γH2AX foci were quantified 1 h, 3 h, and 6 h after IR. The number (**B**) and relative total area (**C**) of γH2AX foci per nucleus. The total area of γH2AX foci induced in cells untreated with NR, 1 h after IR, was taken as 100%. Data are presented as mean ± standard error (SE) (*n* = 200). Statistical analysis of differences between the groups was carried out by one-way ANOVA with post hoc comparisons using Tukey test. *** indicates statistically significant difference at *p* < 0.001. ns indicates that statistical difference is not significant. CTRL marks unirradiated control cells.

Furthermore, we optimized the conditions for induction and detection of DSB in HDF after IR treatment. IR-induced DNA DSB were indirectly monitored using immunocytochemical analysis of histone variant H2AX phosphorylated at Ser139 (γH2AX), the marker of DSB [7]. The cells cultured under standard conditions were exposed to IR at a moderate dose of 1 Gy. Next, control and irradiated cells were fixed, and γH2AX immunofluorescence staining was performed (Figure 2A). γH2AX foci can be detected in the nuclei of S-phase unirradiated cells due to occasional formation of DSB during normal replication [59]. Therefore, to exclude replicating cells from the analysis, we used DNA labeling with a thymidine analogue, 5-ethynyl-2′-deoxyuridine (EdU), which was added to the growth medium 20 min before fixation (Figure S2). G2 cells were also excluded from analysis due to the increased size of the nucleus and specific Ki-67 staining (Figure S2). Figure 2B,C shows the results of quantitative analysis of γH2AX foci in the nuclei of control and irradiated cells in G0 and G1 phases of the cell cycle, 1 h, 3 h, and 6 h after IR exposure. About 30–35 γH2AX foci per nucleus were detected 1 h after IR (Figure 2B). During subsequent incubation, a decrease in the number and the total area of foci per nucleus was observed, which indicates the effective DNA DSB repair in HDF exposed to IR at a dose of 1 Gy. NR at a concentration of 150 µM was added to the cell growth medium to find out how the stimulation of NAD biosynthesis could modulate the capacity of DNA DSB repair. Overall, 24 h after the addition of NR, the cells were irradiated at a dose of 1 Gy and incubated in the growth medium containing NR (at the same concentration). In NR-treated cells, both the number (Figure 2B) and the total area of foci per nucleus (Figure 2C) remained at the level of NR-untreated irradiated cells 1 h, 3 h, and 6 h after IR treatment. Thus, the increase in intracellular NAD level had no effect on the efficiency of formation and elimination of γH2AX foci, i.e., it did not influence DNA DSB repair capacity after IR treatment at a dose of 1 Gy.

### 3.3. IR Does Not Affect NAD Levels in HDF

**Figure 3:**
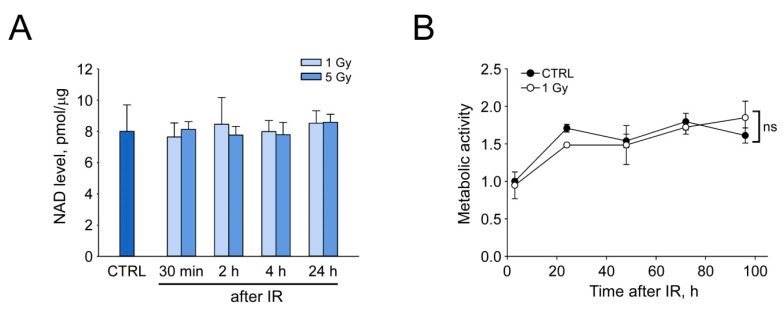
The effect of IR on NAD levels in HDF. HDF were treated with ionizing radiation (IR) at a dose of 1 or 5 Gy and incubated for indicated time intervals in standard culture medium. (**A**) Intracellular NAD level measured by enzymatic colorimetric assay is expressed in picomoles per microgram of total protein in cell extracts. Data are presented as mean ± SD (*n* = 3). (**B**) Relative metabolic activity of cells was assessed using the MTT assay. Metabolic activity of untreated cells (control) was taken as 1. Data are presented as mean ± SD (*n* = 3). Statistical analysis of differences between the groups was carried out by two-way ANOVA with post hoc comparisons using the Tukey test. ns indicates that statistical difference is not significant. CTRL in (**A**,**B**) marks unirradiated control cells.

To elucidate how the exposure to IR can influence the cellular NAD content, we treated HDF with IR at a dose of 1 Gy or 5 Gy and measured NAD level in cell extracts at various time intervals. NAD concentration in control cells was about 8 pmol/µg and remained unchanged at 0.5, 2, 4, and 24 h after IR at both doses (Figure 3A). Furthermore, differences in metabolic activities of NAD(P)H-dependent dehydrogenases estimated by MTT assay were indistinguishable between irradiated and control cells (Figure 3B and Figure S3). Thus, IR at moderate doses does not induce any depletion of NAD in HDF.

### 3.4. Critical NAD Depletion Decreases the Rate of γH2AX Foci Elimination after IR

**Figure 4:**
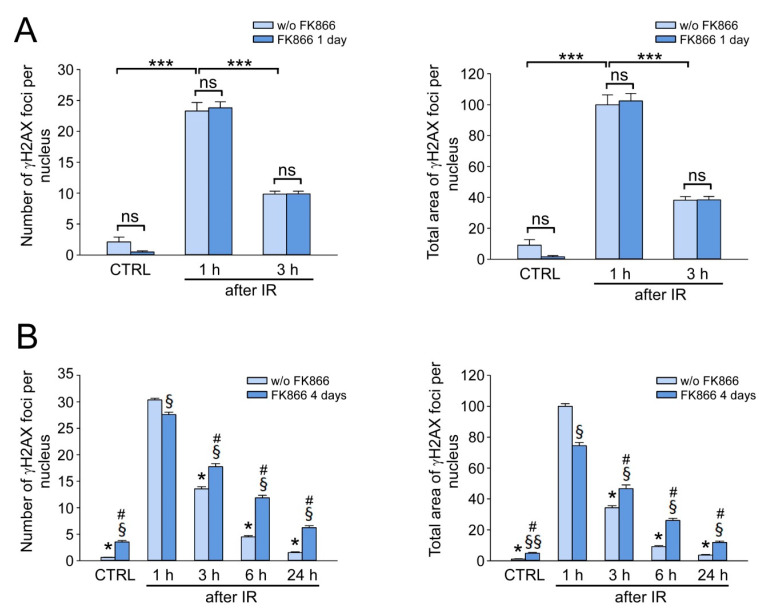
The effect of the inhibition of NAD biosynthesis on γH2AX foci elimination in IR-exposed HDF. HDF were treated with FK866 for 1 day (**A**) or 4 days (**B**) and then irradiated at a dose of 1 Gy (IR). CTRL indicates unirradiated control cells. w/o FK866—marks cells untreated with FK866. Cells were fixed at different time points after IR (as indicated) and stained for γH2AX. The number of γH2AX foci per nucleus (left panels) and relative total area of γH2AX foci per nucleus (right panels) are shown. Total area of γH2AX foci induced in cells untreated with FK866, 1 h after IR, was taken as 100%. Data are presented as mean ± SE (*n* = 200). Statistical analysis of differences between the groups was carried out by one (**A**) or two (**B**)-way ANOVA with post hoc comparisons using Tukey test. In (**A**), *** indicates statistically significant difference at *p* < 0.001. ns—indicates that the difference is not significant. In (**B**), * indicates statistically significant difference at *p* < 0.001 vs. the time point 1 h w/o FK866; §—statistically significant difference at *p* < 0.001 vs. the same time point but w/o FK866; §§—statistically significant difference at *p* < 0.05 vs. the same time point but w/o FK866; #—statistically significant difference at *p* < 0.001 vs. the time point 1 h with FK866.

Next, we intended to clarify how the depletion of the NAD pool by the NAMPT inhibitor, FK866, could affect the efficiency of DNA DSB repair. HDFs were irradiated at a dose of 1 Gy and incubated in the medium containing FK866 for 1 or 4 days, then the number of γH2AX foci and the total area of foci per nucleus were estimated in treated and untreated cells as described in Section 3.2. As a result of a 1-day treatment with FK866, there was a decrease in intracellular NAD level by 50% compared to that in control cells (Figure 1A), but the number and the total area of γH2AX foci per nucleus did not change 1 h and 3 h after IR treatment (Figure 4A). Thus, a mild decrease in the NAD pool did not affect both the induction and the elimination of DNA DSB. After 4 days of treatment with FK866, intracellular NAD was hardly detectable in HDF (Figure 1A). This resulted in less efficient IR-induced γH2AX foci formation—their number per nucleus 1 h after IR was 10% less (Figure 4B, left panel)—and the total area dropped by 25% (Figure 4B, right panel) compared to untreated cells. Surprisingly, despite the almost complete depletion of NAD, the elimination of γH2AX foci was observed at 3, 6, and 24 h after IR, but it was less effective than in FK866-untreated cells (Figure 4B). These results indicated that under conditions of critical NAD depletion, HDF retained the ability to eliminate DNA DSB, but the DSB repair capacity was decreased in comparison to cells with normal NAD levels.

### 3.5. NAD Depletion Suppresses the Accumulation of the Activated Form of ATM Kinase at DSB Sites and Its Colocalization with γH2AX

**Figure 5:**
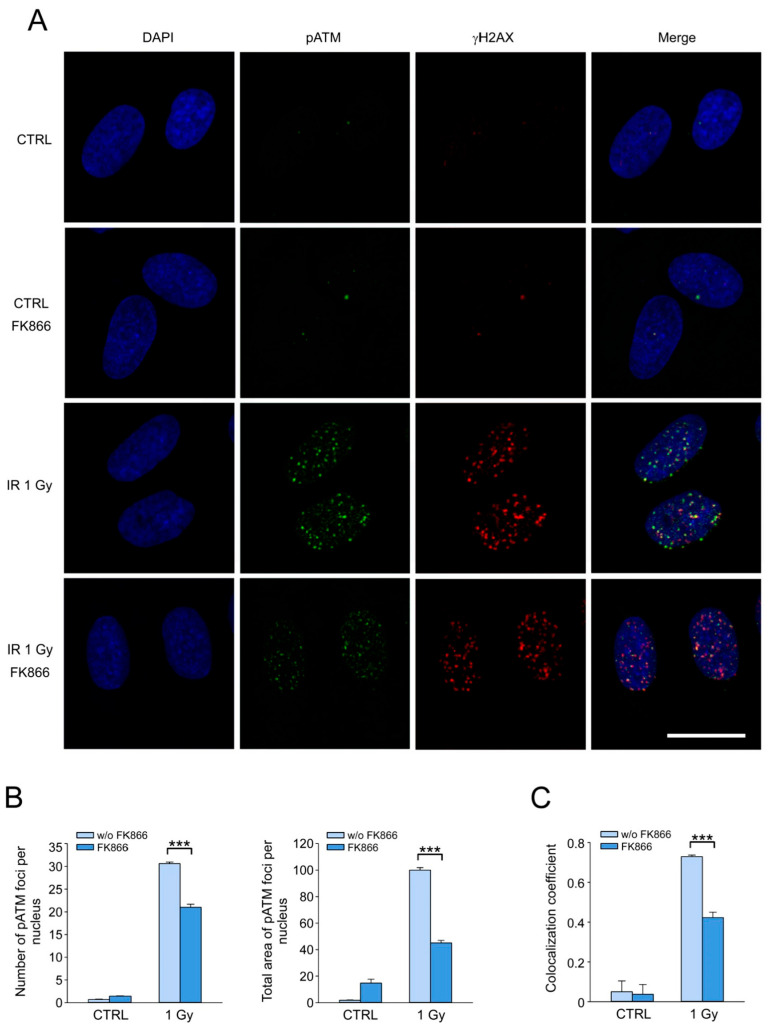
NAD depletion suppresses the accumulation of the activated form of ATM kinase at DSB sites and its colocalization with γH2AX. HDF were treated with FK866 for 4 days, and then irradiated at a dose of 1 Gy (IR). One hour after IR, cells were fixed and stained for γH2AX and ATM kinase phosphorylated at Ser1981 (pATM). (**A**) Immunofluorescence of γH2AX (red) and pATM (green) foci. Cell nuclei were counterstained with DAPI (blue). Scale bar, 20 µm. (**B**) The number (left panel) and relative total area (right panel) of pATM foci per nucleus. Total area of pATM foci induced in cells untreated with FK866, 1 h after IR, was taken as 100%. (**C**) Quantification of colocalization of γH2AX and pATM by Pearson’s correlation. Data are presented as mean ± SE (*n* = 200). Statistical analysis of differences between the groups was carried out by one-way ANOVA with post hoc comparisons using the Tukey test. *** indicates statistically significant difference at *p* < 0.001. CTRL marks unirradiated control cells.

ATM kinase is one of the key regulators of cellular response to the induction of DNA DSB. At sites of DSB, ATM is activated by autophosphorylation at Ser1981 and phosphorylates various substrate proteins, including H2AX [6,8]. Our next objective was to test the hypothesis that decreased DNA DSB repair capacity in NAD-depleted cells might be associated with an impaired activation of ATM kinase at the sites of DNA damage. To do this, we treated cells with IR at a dose of 1 Gy after 4 days of incubation with 2 µM FK866. Cells were fixed 1 h after irradiation, and double immunostaining for γH2AX and activated form of ATM kinase (phosphorylated at Ser1981, pATM) was performed. The cells in G0 and G1 phases of the cell cycle were analyzed. Using confocal microscopy, it was shown that the irradiation of control cells induced the accumulation of pATM foci highly colocalized with γH2AX (Figure 5A). After 4 days of treatment with FK866, we observed a considerable decrease in the number and the total area of pATM foci per nucleus (Figure 5A,B). The degree of colocalization of pATM/γH2AX foci was visually reduced (Figure 5A). For quantitative colocalization analysis of foci in confocal fluorescence microscopy images, Pearson’s correlation coefficient (PCC) was used [53]. Consistent with confocal microscopy visual observations, high values of PCC (more than 0.7) for irradiated control cells indicated a high degree of colocalization of pATM and γH2AX foci. PCC values estimated for FK866-treated irradiated cells were significantly decreased (approx. 0.4), reflecting a much lower degree of colocalization of pATM and γH2AX in NAD-depleted cells (Figure 5C). These results indicate that NAD depletion impairs ATM kinase activation and its colocalization with γH2AX in response to IR.

## 4. Discussion

DSB repair is promoted by PARP and sirtuin family proteins that use NAD as a substrate to catalyze ADP-ribosylation or deacetylation of various targets in response to DSB induction. PARP1 is a major consumer of NAD in mammalian cells under conditions of genotoxic stress. PARP1 is activated by both single- and double-strand breaks of DNA [60] and catalyzes the cleavage of NAD to ADP-ribose and Nam, followed by the transfer of ADP-ribose units to target proteins, including itself [24]. DNA oxidative and DNA alkylating agents, which generate DNA single-strand breaks as intermediates of base excision repair (BER), can induce hyperactivation of PARP1 and massive poly-ADP-ribosylation leading to a dramatic drop of the intracellular NAD, ATP depletion and cell death [61,62,63,64,65,66]. Supplementation of cells with various forms of vitamin B3 replenishes NAD levels and recovers cell viability under genotoxic conditions [65,67], and it is suggested that elevated NAD can promote single-strand break/BER repair [68,69]. Yet, not much is known about how the induction of DSB affects the level of intracellular NAD and how changes in NAD bioavailability can influence DSB repair.

In the present study, we tested how the stimulation and inhibition of NAD biosynthesis changed DSB repair capacity in HDF exposed to moderate doses of IR. First, we demonstrated that the boosting of NAD levels by NR did not change the efficiency of DSB repair (as indicated by γH2AX foci elimination) after the exposure of cells to IR at a dose of 1 Gy. Moreover, IR, even at a dose of 5 Gy, did not lead to any changes in intracellular NAD concentration. Similar results were obtained in the previous study by Weidele et al. on peripheral blood mononuclear cells. Irradiation with X-ray at a dose of 2.5 or 5 Gy led to only a slight decrease in NAD levels, whereas the treatment with NAD precursor, nicotinic acid, had no effect on the DNA break repair as revealed by fluorimetric alkaline DNA unwinding assay. A significant (by 50%) drop in NAD was observed only after the exposure of cells to X-ray at a dose of 25 Gy. In this case, the treatment with nicotinic acid slightly increased DNA repair efficiency [70]. Interestingly, in another study, very high doses (50 Gy and above) of IR also caused NAD depletion in several mouse and human cancer cell lines. Moreover, replenishment of NAD levels by treatment of cells with 3 mM Nam improved DSB repair capacity [71]. Thus, our and other results suggest that, under conditions of moderate IR-induced genotoxic stress, the activation of PARP1 and other NAD-dependent enzymes (e.g., PARP2, PARP3, SIRT1, SIRT6, and SIRT7) involved in DNA damage response does not change the concentration of NAD, and it remains optimal for the efficient repair of DSB. The positive effect of NAD boosting on DSB repair can be achieved when IR induces a substantial decrease in intracellular NAD levels, and this requires exposure to very high doses of radiation.

Next, we tested how an imposed depletion of NAD can affect the efficiency of DSB repair in HDF after IR exposure. To do this, we suppressed NAD biosynthesis by FK866, an inhibitor of NAMPT, catalyzing the first step of NAD generation from Nam [55]. We showed that the decrease in intracellular NAD by 50% after 1 day of treatment with FK866 did not affect the DSB repair capacity. Moreover, even after 4 days of FK866 treatment, when intracellular NAD was almost undetectable, cells were still able to eliminate IR-induced DNA DSB, although DSB repair capacity was reduced compared to cells with normal NAD levels. Specifically, we observed a decrease in both number and total area of γH2AX foci per nucleus 1 h after a moderate (1 Gy) IR exposure under conditions of critical depletion of NAD. On the contrary, the kinetics of the subsequent elimination of γH2AX foci was significantly slowed down without NAD in comparison to control cells.

ATM-dependent phosphorylation of H2AX at serine 139, forming γH2AX foci at the sites of DSB, is one of the key events in the early cell response to DSB [7,8]. The NAD-dependent enzymes PARP1 and SIRT1 promote the recruitment and activation (autophosphorylation) of ATM at DSB [27,28,38]. Moreover, PARP1- and SIRT1-deficient cells display an altered activation of ATM and a reduced γH2AX foci formation in response to DSB-inducing agents [28,38]. Therefore, we hypothesized that the reduced γH2AX foci formation in response to IR, followed by the suppressed elimination of these foci in NAD-depleted cells, might be caused by the impaired activation and recruitment of ATM to DSB sites. In support of this assumption, we showed that IR-induced accumulation of the activated form of ATM at DSB sites and its colocalization with γH2AX were significantly decreased in HDF pretreated with FK866.

Since the homologous recombination is not available in G0- and G1-phase cells that were selected for the DSB repair analysis, we can assume that the maintenance of physiological concentrations of NAD is necessary for the efficient NHEJ, which is the major DSB repair pathway in G0/G1-phase. However, the very fact of DSB repair in cells lacking NAD indicates that NAD-dependent processes, such as protein deacetylation and ADP-ribosylation, may be important but not crucial factors controlling the NHEJ of DSB induced by moderate doses of IR.

## Data Availability

The data presented in this study are available from the corresponding authors upon request.

## Supplementary Materials

The following supporting information can be downloaded at: https://www.mdpi.com/article/10.3390/cells12111518/s1, Figure S1: Modulation of NAD biosynthesis in cultured human dermal fibroblasts, Figure S2: Selection of G0/G1 IR-exposed HDF for γH2AX foci quantification, and Figure S3: Metabolic activity of IR-exposed HDF.

## References

[1] Ciccia A., Elledge S.J. (2010). The DNA damage response: Making it safe to play with knives. Mol. Cell.

[2] Jasin M., Rothstein R. (2013). Repair of strand breaks by homologous recombination. Cold Spring Harb. Perspect. Biol..

[3] Chang H.H.Y., Pannunzio N.R., Adachi N., Lieber M.R. (2017). Non-homologous DNA end joining and alternative pathways to double-strand break repair. Nat. Rev. Mol. Cell Biol..

[4] Bakkenist C.J., Kastan M.B. (2003). DNA damage activates ATM through intermolecular autophosphorylation and dimer dissociation. Nature.

[5] Lee J.H., Paull T.T. (2005). ATM activation by DNA double-strand breaks through the Mre11-Rad50-Nbs1 complex. Science.

[6] Shiloh Y., Ziv Y. (2013). The ATM protein kinase: Regulating the cellular response to genotoxic stress, and more. Nat. Rev. Mol. Cell Biol..

[7] Rogakou E.P., Pilch D.R., Orr A.H., Ivanova V.S., Bonner W.M. (1998). DNA double-stranded breaks induce histone H2AX phosphorylation on serine 139. J. Biol. Chem..

[8] Burma S., Chen B.P., Murphy M., Kurimasa A., Chen D.J. (2001). ATM phosphorylates histone H2AX in response to DNA double-strand breaks. J. Biol. Chem..

[9] Furuta T., Takemura H., Liao Z.Y., Aune G.J., Redon C., Sedelnikova O.A., Pilch D.R., Rogakou E.P., Celeste A., Chen H.T. (2003). Phosphorylation of histone H2AX and activation of Mre11, Rad50, and Nbs1 in response to replication-dependent DNA double-strand breaks induced by mammalian DNA topoisomerase I cleavage complexes. J. Biol. Chem..

[10] Stiff T., O’Driscoll M., Rief N., Iwabuchi K., Lobrich M., Jeggo P.A. (2004). ATM and DNA-PK function redundantly to phosphorylate H2AX after exposure to ionizing radiation. Cancer Res..

[11] Ward I.M., Chen J. (2001). Histone H2AX is phosphorylated in an ATR-dependent manner in response to replicational stress. J. Biol. Chem..

[12] Iacovoni J.S., Caron P., Lassadi I., Nicolas E., Massip L., Trouche D., Legube G. (2010). High-resolution profiling of gammaH2AX around DNA double strand breaks in the mammalian genome. EMBO J..

[13] Rogakou E.P., Boon C., Redon C., Bonner W.M. (1999). Megabase chromatin domains involved in DNA double-strand breaks in vivo. J. Cell Biol..

[14] Scully R., Xie A. (2013). Double strand break repair functions of histone H2AX. Mutat. Res..

[15] Sedelnikova O.A., Rogakou E.P., Panyutin I.G., Bonner W.M. (2002). Quantitative detection of (125)IdU-induced DNA double-strand breaks with gamma-H2AX antibody. Radiat. Res..

[16] Rothkamm K., Lobrich M. (2003). Evidence for a lack of DNA double-strand break repair in human cells exposed to very low X-ray doses. Proc. Natl. Acad. Sci. USA.

[17] Firsanov D.V., Kulikova V.A., Solovjeva L.V., Mikhailov V.M., Nikiforov A.A., Svetlova M.P. (2021). Methods for the detection of DNA damage. Genome Stability: From Virus to Human Application.

[18] Kulikova V.A., Gromyko D.V., Nikiforov A.A. (2018). The Regulatory Role of NAD in Human and Animal Cells. Biochemistry.

[19] Xie N., Zhang L., Gao W., Huang C., Huber P.E., Zhou X., Li C., Shen G., Zou B. (2020). NAD^+^ metabolism: Pathophysiologic mechanisms and therapeutic potential. Signal Transduct. Target. Ther..

[20] Yang Y., Sauve A.A. (2016). NAD^+^ metabolism: Bioenergetics, signaling and manipulation for therapy. Biochim. Biophys. Acta.

[21] Lagunas-Rangel F.A. (2019). Current role of mammalian sirtuins in DNA repair. DNA Repair.

[22] Beck C., Robert I., Reina-San-Martin B., Schreiber V., Dantzer F. (2014). Poly(ADP-ribose) polymerases in double-strand break repair: Focus on PARP1, PARP2 and PARP3. Exp. Cell Res..

[23] Yang G., Liu C., Chen S.H., Kassab M.A., Hoff J.D., Walter N.G., Yu X. (2018). Super-resolution imaging identifies PARP1 and the Ku complex acting as DNA double-strand break sensors. Nucleic Acids Res..

[24] Ray Chaudhuri A., Nussenzweig A. (2017). The multifaceted roles of PARP1 in DNA repair and chromatin remodelling. Nat. Rev. Mol. Cell Biol..

[25] Haince J.F., McDonald D., Rodrigue A., Dery U., Masson J.Y., Hendzel M.J., Poirier G.G. (2008). PARP1-dependent kinetics of recruitment of MRE11 and NBS1 proteins to multiple DNA damage sites. J. Biol. Chem..

[26] Haince J.F., Kozlov S., Dawson V.L., Dawson T.M., Hendzel M.J., Lavin M.F., Poirier G.G. (2007). Ataxia telangiectasia mutated (ATM) signaling network is modulated by a novel poly(ADP-ribose)-dependent pathway in the early response to DNA-damaging agents. J. Biol. Chem..

[27] Goodarzi A.A., Lees-Miller S.P. (2004). Biochemical characterization of the ataxia-telangiectasia mutated (ATM) protein from human cells. DNA Repair.

[28] Aguilar-Quesada R., Munoz-Gamez J.A., Martin-Oliva D., Peralta A., Valenzuela M.T., Matinez-Romero R., Quiles-Perez R., Menissier-de Murcia J., de Murcia G., Ruiz de Almodovar M. (2007). Interaction between ATM and PARP-1 in response to DNA damage and sensitization of ATM deficient cells through PARP inhibition. BMC Mol. Biol..

[29] Li M., Yu X. (2013). Function of BRCA1 in the DNA damage response is mediated by ADP-ribosylation. Cancer Cell.

[30] Galande S., Kohwi-Shigematsu T. (1999). Poly(ADP-ribose) polymerase and Ku autoantigen form a complex and synergistically bind to matrix attachment sequences. J. Biol. Chem..

[31] Spagnolo L., Barbeau J., Curtin N.J., Morris E.P., Pearl L.H. (2012). Visualization of a DNA-PK/PARP1 complex. Nucleic Acids Res..

[32] Ruscetti T., Lehnert B.E., Halbrook J., Le Trong H., Hoekstra M.F., Chen D.J., Peterson S.R. (1998). Stimulation of the DNA-dependent protein kinase by poly(ADP-ribose) polymerase. J. Biol. Chem..

[33] Caron M.C., Sharma A.K., O’Sullivan J., Myler L.R., Ferreira M.T., Rodrigue A., Coulombe Y., Ethier C., Gagne J.P., Langelier M.F. (2019). Poly(ADP-ribose) polymerase-1 antagonizes DNA resection at double-strand breaks. Nat. Commun..

[34] Luijsterburg M.S., de Krijger I., Wiegant W.W., Shah R.G., Smeenk G., de Groot A.J.L., Pines A., Vertegaal A.C.O., Jacobs J.J.L., Shah G.M. (2016). PARP1 Links CHD2-Mediated Chromatin Expansion and H3.3 Deposition to DNA Repair by Non-homologous End-Joining. Mol. Cell.

[35] Rulten S.L., Fisher A.E., Robert I., Zuma M.C., Rouleau M., Ju L., Poirier G., Reina-San-Martin B., Caldecott K.W. (2011). PARP-3 and APLF function together to accelerate nonhomologous end-joining. Mol. Cell.

[36] Beck C., Boehler C., Guirouilh Barbat J., Bonnet M.E., Illuzzi G., Ronde P., Gauthier L.R., Magroun N., Rajendran A., Lopez B.S. (2014). PARP3 affects the relative contribution of homologous recombination and nonhomologous end-joining pathways. Nucleic Acids Res..

[37] Toiber D., Erdel F., Bouazoune K., Silberman D.M., Zhong L., Mulligan P., Sebastian C., Cosentino C., Martinez-Pastor B., Giacosa S. (2013). SIRT6 recruits SNF2H to DNA break sites, preventing genomic instability through chromatin remodeling. Mol. Cell.

[38] Dobbin M.M., Madabhushi R., Pan L., Chen Y., Kim D., Gao J., Ahanonu B., Pao P.C., Qiu Y., Zhao Y. (2013). SIRT1 collaborates with ATM and HDAC1 to maintain genomic stability in neurons. Nat. Neurosci..

[39] Wang R.H., Sengupta K., Li C., Kim H.S., Cao L., Xiao C., Kim S., Xu X., Zheng Y., Chilton B. (2008). Impaired DNA damage response, genome instability, and tumorigenesis in SIRT1 mutant mice. Cancer Cell.

[40] Yuan Z., Zhang X., Sengupta N., Lane W.S., Seto E. (2007). SIRT1 regulates the function of the Nijmegen breakage syndrome protein. Mol. Cell.

[41] Jeong J., Juhn K., Lee H., Kim S.H., Min B.H., Lee K.M., Cho M.H., Park G.H., Lee K.H. (2007). SIRT1 promotes DNA repair activity and deacetylation of Ku70. Exp. Mol. Med..

[42] Li K., Casta A., Wang R., Lozada E., Fan W., Kane S., Ge Q., Gu W., Orren D., Luo J. (2008). Regulation of WRN protein cellular localization and enzymatic activities by SIRT1-mediated deacetylation. J. Biol. Chem..

[43] Lin Y.H., Yuan J., Pei H., Liu T., Ann D.K., Lou Z. (2015). KAP1 Deacetylation by SIRT1 Promotes Non-Homologous End-Joining Repair. PLoS ONE.

[44] Mao Z., Hine C., Tian X., Van Meter M., Au M., Vaidya A., Seluanov A., Gorbunova V. (2011). SIRT6 promotes DNA repair under stress by activating PARP1. Science.

[45] Onn L., Portillo M., Ilic S., Cleitman G., Stein D., Kaluski S., Shirat I., Slobodnik Z., Einav M., Erdel F. (2020). SIRT6 is a DNA double-strand break sensor. eLife.

[46] McCord R.A., Michishita E., Hong T., Berber E., Boxer L.D., Kusumoto R., Guan S., Shi X., Gozani O., Burlingame A.L. (2009). SIRT6 stabilizes DNA-dependent protein kinase at chromatin for DNA double-strand break repair. Aging.

[47] Vazquez B.N., Thackray J.K., Serrano L. (2017). Sirtuins and DNA damage repair: SIRT7 comes to play. Nucleus.

[48] Vazquez B.N., Thackray J.K., Simonet N.G., Kane-Goldsmith N., Martinez-Redondo P., Nguyen T., Bunting S., Vaquero A., Tischfield J.A., Serrano L. (2016). SIRT7 promotes genome integrity and modulates non-homologous end joining DNA repair. EMBO J..

[49] Tang M., Li Z., Zhang C., Lu X., Tu B., Cao Z., Li Y., Chen Y., Jiang L., Wang H. (2019). SIRT7-mediated ATM deacetylation is essential for its deactivation and DNA damage repair. Sci. Adv..

[50] Kill I.R. (1996). Localisation of the Ki-67 antigen within the nucleolus. Evidence for a fibrillarin-deficient region of the dense fibrillar component. J. Cell Sci..

[51] Sales Gil R., Vagnarelli P. (2018). Ki-67: More Hidden behind a ‘Classic Proliferation Marker’. Trends Biochem. Sci..

[52] Solovjeva L., Firsanov D., Vasilishina A., Chagin V., Pleskach N., Kropotov A., Svetlova M. (2015). DNA double-strand break repair is impaired in presenescent Syrian hamster fibroblasts. BMC Mol. Biol..

[53] Dunn K.W., Kamocka M.M., McDonald J.H. (2011). A practical guide to evaluating colocalization in biological microscopy. Am. J. Physiol. Cell Physiol..

[54] Nikiforov A., Kulikova V., Ziegler M. (2015). The human NAD metabolome: Functions, metabolism and compartmentalization. Crit. Rev. Biochem. Mol. Biol..

[55] Hasmann M., Schemainda I. (2003). FK866, a highly specific noncompetitive inhibitor of nicotinamide phosphoribosyltransferase, represents a novel mechanism for induction of tumor cell apoptosis. Cancer Res..

[56] Bieganowski P., Brenner C. (2004). Discoveries of nicotinamide riboside as a nutrient and conserved NRK genes establish a Preiss-Handler independent route to NAD+ in fungi and humans. Cell.

[57] Cercillieux A., Ciarlo E., Canto C. (2022). Balancing NAD^+^ deficits with nicotinamide riboside: Therapeutic possibilities and limitations. Cell. Mol. Life Sci..

[58] Yoshino J., Baur J.A., Imai S.I. (2018). NAD^+^ Intermediates: The Biology and Therapeutic Potential of NMN and NR. Cell Metab..

[59] MacPhail S.H., Banath J.P., Yu Y., Chu E., Olive P.L. (2003). Cell cycle-dependent expression of phosphorylated histone H2AX: Reduced expression in unirradiated but not X-irradiated G1-phase cells. Radiat. Res..

[60] Alemasova E.E., Lavrik O.I. (2019). Poly(ADP-ribosyl)ation by PARP1: Reaction mechanism and regulatory proteins. Nucleic Acids Res..

[61] Berger N.A., Sims J.L., Catino D.M., Berger S.J. (1983). Poly(ADP-ribose) polymerase mediates the suicide response to massive DNA damage: Studies in normal and DNA-repair defective cells. Princess Takamatsu Symp..

[62] Wilk A., Hayat F., Cunningham R., Li J., Garavaglia S., Zamani L., Ferraris D.M., Sykora P., Andrews J., Clark J. (2020). Extracellular NAD^+^ enhances PARP-dependent DNA repair capacity independently of CD73 activity. Sci. Rep..

[63] Fouquerel E., Goellner E.M., Yu Z., Gagne J.P., Barbi de Moura M., Feinstein T., Wheeler D., Redpath P., Li J., Romero G. (2014). ARTD1/PARP1 negatively regulates glycolysis by inhibiting hexokinase 1 independent of NAD^+^ depletion. Cell Rep..

[64] Nishida T., Naguro I., Ichijo H. (2022). NAMPT-dependent NAD^+^ salvage is crucial for the decision between apoptotic and necrotic cell death under oxidative stress. Cell Death Discov..

[65] Yang Y., Mohammed F.S., Zhang N., Sauve A.A. (2019). Dihydronicotinamide riboside is a potent NAD^+^ concentration enhancer in vitro and in vivo. J. Biol. Chem..

[66] Ha H.C., Snyder S.H. (1999). Poly(ADP-ribose) polymerase is a mediator of necrotic cell death by ATP depletion. Proc. Natl. Acad. Sci. USA.

[67] Formentini L., Moroni F., Chiarugi A. (2009). Detection and pharmacological modulation of nicotinamide mononucleotide (NMN) in vitro and in vivo. Biochem. Pharmacol..

[68] Koczor C.A., Saville K.M., Andrews J.F., Clark J., Fang Q., Li J., Al-Rahahleh R.Q., Ibrahim M., McClellan S., Makarov M.V. (2021). Temporal dynamics of base excision/single-strand break repair protein complex assembly/disassembly are modulated by the PARP/NAD^+^/SIRT6 axis. Cell Rep..

[69] Saville K.M., Clark J., Wilk A., Rogers G.D., Andrews J.F., Koczor C.A., Sobol R.W. (2020). NAD^+^-mediated regulation of mammalian base excision repair. DNA Repair.

[70] Weidele K., Beneke S., Burkle A. (2017). The NAD^+^ precursor nicotinic acid improves genomic integrity in human peripheral blood mononuclear cells after X-irradiation. DNA Repair.

[71] Riklis E., Kol R., Marko R. (1990). Trends and developments in radioprotection: The effect of nicotinamide on DNA repair. Int. J. Radiat. Biol..

