# Supplementary material for: The Impact of NAD Bioavailability on DNA Double-Strand Break Repair Capacity in Human Dermal Fibroblasts after Ionizing Radiation
Source: Cells. 2023 May 31;12(11):1518. doi: 10.3390/cells12111518 (PMC10252650; doi:10.3390/cells12111518)
Supplement: Supplementary file 1 [file cells-12-01518-s001.zip › cells-2406101-supplementary.pdf]

Supplementary Figures

Figure S1

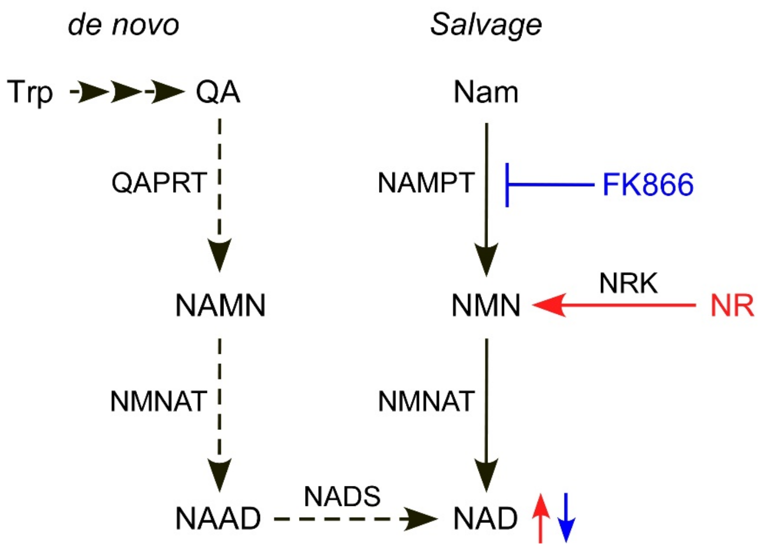

Figure S1. Modulation of NAD biosynthesis in cultured human dermal fibroblasts (HDF). Human dermal fibroblasts (HDF) cultured in standard medium can synthesize NAD from the pyridine base nicotinamide (Nam), form of vitamin B3, via the salvage pathway or from tryptophan (Trp) via the *de novo* pathway (dashed arrows). Nam is converted by the Nam phosphoribosyltransferase (NAMPT) to the Nam mononucleotide (NMN), which in turn is adenylated by the NMN adenylyltransferases (NMNAT) to form NAD. Trp is converted to quinolinic acid (QA), which is a substrate of QA phosphoribosyltransferase (QAPRT) that generates nicotinic acid mononucleotide (NAMN). NAMN is converted to nicotinic acid adenine dinucleotide (NAAD) by NMNAT. NAD synthetase (NADS) amidates NAAD to NAD. Suppression of NAD synthesis from Nam by specific NAMPT inhibitor, FK866, leads to NAD depletion (highlighted in blue). Intracellular NAD levels can be increased by nicotinamide riboside (NR) which stimulates an alternative NAD biosynthetic pathway via its phosphorylation to NMN by the NR kinases (NRK) (highlighted in red).

Figure S2

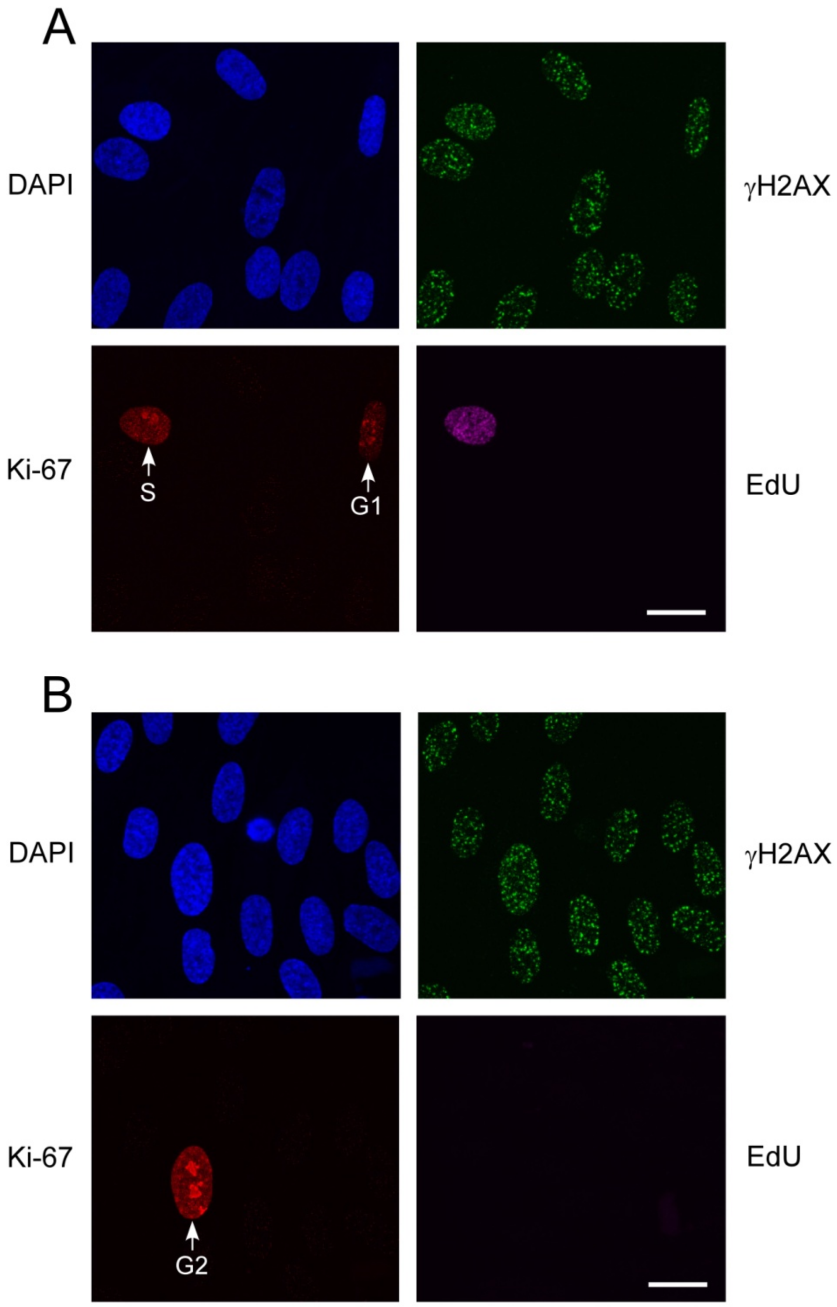

Figure S2. Characteristic staining of IR-exposed HDF for γH2AX, Ki-67 and EdU. After 5-ethynyl-2'-deoxyuridine (EdU) incorporation, HDF were treated with ionizing radiation (IR) at a dose of 1 Gy. 1 h after IR, cells were fixed and stained for γH2AX, Ki-67 and EdU, cell nuclei were stained with DAPI. Maximum intensity projections of confocal sections are presented. **(A)** Characteristic staining of S- and G1-phase cells for Ki-67 and S-phase cells for EdU. **(B)** Characteristic staining of G2-phase cells for Ki-67. Scale bar, 20 μm.

Figure S3

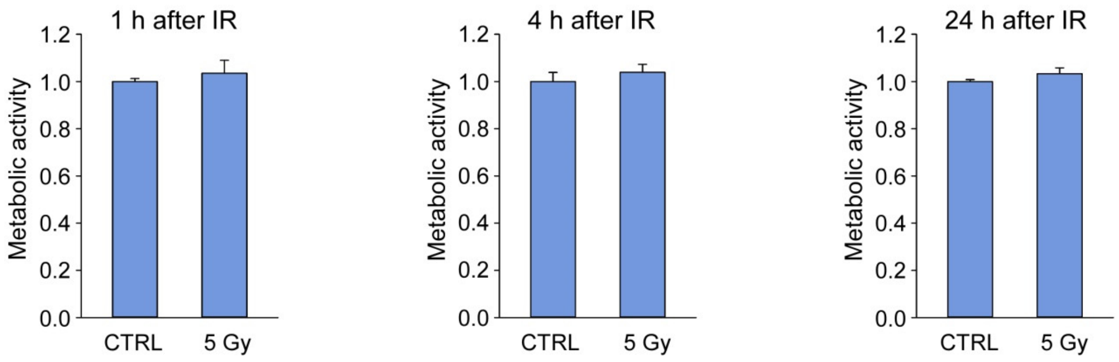

Figure S3. Metabolic activity of IR-exposed HDF. Cells were exposed to IR at a dose of 5 Gy using the X-ray irradiator RAP-150/300-14. 1 h, 4 h or 24 h after IR, relative metabolic activity was determined using the MTT assay. Metabolic activity of non-irradiated control cells (CTRL) was taken as 1. Data are presented as mean +/- standard deviation (n=3).
